# 

**Published:** 1857-04

**Authors:** 


### CONTENTS.

ORIGINAL COMMUNICATIONS— PAGE.

Etiology of Milksickness: an Inaugural Thesis. By G. W. Wilkinson, 151

A very remarkable Case of Valvular Disease of the Heart, resulting in absolute Death of a Portion of the Inferior Extremities. By F. R. Payne........ 159

Injuries of the Knee-Joint. By Benjamin Woodward........ 162

Clinical Cases treated in the Surgical Wards of the Mercy Hospital..... 163

SELECTIONS—

Editorial Correspondence of Atlanta Medical and Surgical Journal...... 166

Dr. Edward Brown-Sequard's Experimental and Clinical Researches, Epilepsy, (Continued)........ 175

BOOK NOTICES—

Transactions of the American Medical Association, Vol. IX. 1856........ 178

Rigby on the Constitutional Treatment of Female Diseases........ 185

EDITORIAL—

Death of Dr. Kane........ 186

Delegates to the American Medical Association........ 188

Annual Meeting of the Illinois State Medical Society........ 189

Agent........ 189

Medical Controversy........ 190

The Influence of Alcohol on Tuberculosis........ 190

To Correspondents........ 196

Iodide of Zinc as a Local Application........ 196

Editorial Change........ 197

MISCELLANEOUS ITEMS—

Iodine in Vomiting in Pregnancy, by Dr. Eulenberg........ 197

Dispersion of Milk........ 198

Mortality of Women in Childbirth........ 198

